# Primary Healthcare Case Management Nurses and Assistance Provided to Chronic Patients: A Narrative Review

**DOI:** 10.3390/healthcare12111054

**Published:** 2024-05-22

**Authors:** María José Molina-Gil, María Dolores Guerra-Martín, Rocío De Diego-Cordero

**Affiliations:** 1 South Seville Health Management Area, Andalusian Health Service, 41071 Seville, Spain; mmolina3@us.es; 2 Francisco Maldonado University Center of Osuna, 41640 Seville, Spain; 3 Faculty of Nursing, Physiotherapy and Podiatry, University of Seville, 41009 Seville, Spain; rdediego2@us.es; 4 Institute of Biomedicine of Seville (IBiS), 41013 Seville, Spain

**Keywords:** case managers, chronic disease, primary care nursing, review

## Abstract

Population aging is causing increases in the numbers of chronic diseases, with the consequent need for changes in health systems to better assist patients with chronic conditions. A narrative review was conducted in this study with the objective of analysing the scientific evidence on the care and assistance provided by Case Management Nurses (CMNs) to chronic patients in primary healthcare. A total of 15 articles published in English, Spanish, and Portuguese were selected in the following databases: PubMed, Embase, Cochrane Library, Scopus, Dialnet, Cinahl, and Web of Science. In total, 46.6% of the studies showed the assistance provided by CMNs for chronic pathologies. Most of the articles selected (80%) considered that the assistance offered by case management nurses in relation to chronic diseases is effective, enabling cost reductions, which supposes benefits at the economic and political levels. It was concluded that CMNs have proven to be efficient in caring for people with chronic diseases, improving the quality of life of these people and their caregivers; therefore, they have a fundamental role in the PHC.

## 1. Introduction

Population aging is a major concern in terms of politics, citizenship, and health. There is an ongoing increase in expenses at the health level, especially in the consumption of medications and in hospital admissions. People over the age of 65 consume more than 50% of the medications sold; 80% of older adults take at least one medication a day and 45% are polymedicated, requiring more than three medications a day [1,2,3]. In addition, this population segment is generating ever-increasing expenses in drugs and health resources (more consultations and hospital admissions), ranking first in the United States in the consumption of medications at the global level, followed by Spain [2,3].

Chronic diseases are those with a prolonged evolution and multiple aetiologies that are not solved, causing a social burden both due to economic issues and dependence and disabling problems. Cardiac pathologies, stroke episodes, cancer, diabetes, and chronic respiratory diseases stand out among these ailments; they are responsible for approximately 74% of all deaths worldwide [4,5]. Approximately 41 million people die due to chronic diseases every year, and around 17 million do so before the age of 70. Among chronic diseases, those affecting the cardiovascular system cause 17.9 million deaths per year, followed by tumours (9.3 millions), chronic respiratory diseases (4.1 millions), and diabetes (2 million deaths, including those due to kidney diseases as a result of diabetes) [4,5]. Spain is locus to an increase in the number of chronic diseases and, consequently, to higher health-related expenses [6].

Chronic patients in complex situations are diagnosed with several chronic diseases, present a high vulnerability, and require comprehensive assistance due to the need for long-term, customised, and coordinated care [7,8]. These patients are characterised by using health services to a large extent, especially at the level of urgency services, and they normally require hospitalisation. In addition, they usually have increased dependence levels and are polymedicated; this is associated with increases in the risks of pharmacological interactions and related adverse effects, prescription and intake errors, deficient compliance, hospital readmissions, and mortality. Advanced age, loneliness due to lack of family support, and a high risk of falls are added to the aforementioned [9].

Chronic diseases also affect children and adolescents; therefore, it becomes indispensable for interventions targeted at families and communities to start as early as during PHC, in addition to the promotion of healthy lifestyles [10]. In this sense, taking into account that these ailments affect people of any age, previous studies point to the need to plan changes in health systems in order to better treat chronic diseases with actions such as improved accessibility, continuity, and more comprehensive assistance that considers lifestyles, with periodic physical activity and the suitable intake of certain nutrients standing out, as both can reduce the number of chronic diseases or mitigate the progression of some illnesses [11,12,13].

Chronic diseases have become one of the major epidemics at the global level; this is the result of various factors, such as increased life expectancy, lifestyle changes, and dietary habits. The importance of advanced practice lies in improving the assistance provided to chronic patients, standing out among alternative care methods [14]. We can highlight Case Management (CM) among them. This is a care modality targeted at people with high-intensity care needs, directed both at patients and at caregivers, with the objective of improving care continuity through multidisciplinary and interlevel coordination and integration [15].

Currently, Advanced Practice Nurse (APN) profiles with high professional competence have already been developed at the international level in countries such as the USA, the United Kingdom, Australia, and Canada, achieving reductions in mortality and readmission rates and improvements in quality of life [16]. The Autonomous Community of the Canary Islands (Spain) was a pioneer in implementing CM with the title of Liaison Nurse; this was expanded to other autonomous communities (Andalusia, Catalonia, Basque Country, Valencia, and Madrid). According to the Andalusian Health Quality Agency, CM has been considered as an APN in Andalusia (south of Spain); however, despite formal recognition of this figure in the assistance provided to patients, there is significant heterogeneity in the results from each autonomous community [17,18].

Initially known as Liaison Nurses and later designated as CMNs (both names refer to the same professional profile), the CMN figure was implemented in Andalusia based on Decree No. 137/2002 of Support for Andalusian Families [19]. The CM services were expanded in 2009, including people with complex chronic problems as the target population, thus easing their access to healthcare, multidisciplinary interventions, care continuity guarantees, improved assistance provided to each person and their environment, and ensuring the protection of the patients’ rights and those of their care environments [20].

CM APNs are characterised for presenting a professional profile that provides health services and interventions enhanced with advanced capabilities that exert an influence on clinical health results. APNs have acquired a specialised knowledge base, skills in complex decision making, and clinical competencies for nurses’ expanded performance [18,19,20,21].

CM has evolved favourably and been considered as AP, showing a reduction in costs and improvements in care management [22,23]. In the Primary Health Care Strategy of the Andalusian 2020–2022 Strategic Plan, it was proposed to implement proactive follow-ups of complex chronic patients, prioritising the assistance provided to people with heart failure and Chronic Obstructive Pulmonary Disease (COPD) by the means of instruments that ease continuity and proactive follow-ups by the primary healthcare (PHC) team. The objective of this strategy is to improve quality of life, preventive assistance, and care customisation, as well as to decongest PHC and hospital care urgencies and reduce the number of hospital admissions/readmissions. In this Strategic Plan, the role of CM APNs is reinforced both in relation to people with complex chronic pathologies and in the home environment [24].

Given all of the aforementioned, the objective is to analyse the scientific evidence about the care and assistance provided by CMNs to chronic patients in PHC.

## 2. Materials and Methods

A narrative review of the scientific literature published between December 2022 and January 2023 was carried out following the recommendations set forth in the Cochrane Manual [25] and some of the indications included in the PRISMA report [26]. The following databases were consulted: PubMed, Embase, Cochrane Library, Scopus, Cinahl, Web of Science, and Dialnet.

The search strategy was as follows: chronic* AND (“liaison nurs*” OR “nurse liaison” OR “nurse case manager”) AND (“primary health care” OR “Primary Healthcare” OR “Primary Care”). The term Liaison Nurse (“liaison nurs*”, “nurse liaison”) was included, as this was the name initially given to CMNs (“Case Management Nurses”), corresponding to the same competencies and professional role.

The inclusion criteria were as follows: quantitative and/or qualitative research studies; reviews (bibliographic, systematic, and/or meta-analyses); materials related to case-management/liaison nurses and chronicity in PHC; and articles published in English, Spanish, and Portuguese. No time limits were applied in the searches. The exclusion criterion corresponded to studies that failed to present results, such as projects or programs.

In the first screening step, the eligible studies according to title and abstract were selected; subsequently, all duplicates were removed. Following the selection stage, the second screening was performed by the means of a critical full reading of the articles selected, taking into account agreement and applying the inclusion and exclusion criteria.

The data from the studies were extracted establishing the following elements: 1. Author, Year, and Locus; 2. Study: Type, Methodology, and Objective; 3. Sample (N), Gender, Age, and Period; and 4. Main findings. These data were included in a table following the recommendations provided by Del Pino et al. [27]. The data analysis was conducted through frequencies and percentages.

**Figure 1:**
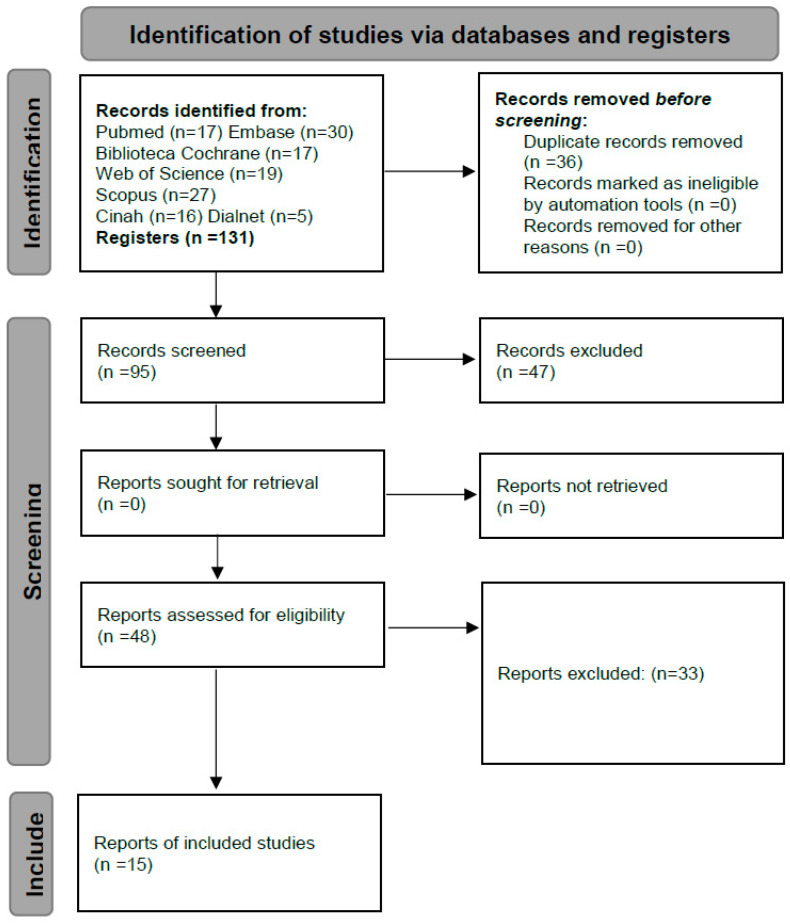
Flowchart corresponding to the studies selected.

A total of 131 studies were initially identified, of which only 15 were finally selected (Figure 1).

## 3. Results

### 3.1. Characteristics of the Studies

Among the studies selected, one of them employed a mixed methodology [28], two were qualitative [29,30], three were systematic reviews [31,32,33], and nine were quantitative studies [34,35,36,37,38,39,40,41,42]. Table 1 presents the characteristics of the studies selected.

### 3.2. Assistance Provided by CMNs to Chronic Patients and Caregivers in PHC

In total, 46.6% (N = 7) of the studies [30,32,33,34,37,41,42] evidenced the assistance provided by CMNs in the case of certain chronic pathologies, such as diabetes [33]; diabetes and arterial hypertension [41]; COPD [34]; diabetes, COPD, and coronary diseases [32]; and diabetes and behavioural health problems [30].

One study made a reference to assistance provided to women with chronic conditions in situations of vulnerability and frailty [35]. In turn, another one dealt with medically complex and frail children and young individuals with chronic health problems [38].

A mixed-methods study related the CM intervention in patients with chronic diseases to psychological distress and activation of the patients [28].

Twenty percent (N = 3) of the studies focused on care provided in urgency services and hospital admissions, relating these aspects to the satisfaction of the patients that had been assisted by CMNs [31,36,40].

Twenty percent (N = 3) of the studies selected [28,31,38] made specific references to the (family) caregivers of patients with chronic pathologies and the assistance they had received from CMNs.

### 3.3. CMNs’ Competencies and Effectiveness

Twenty percent (N = 2) of the articles selected considered that suitable training and certain professional competencies are necessary to hold CMN positions in relation to chronicity. The need for professional experience was also highlighted [31,33]. A clinical trial valued the effectiveness of a care model with the intervention of in-hospital Liaison Nurses and the PHC team [39].

The assistance provided by CMNs to chronic patients via telephone calls clearly proved to be effective in two studies (N = 2) [30,34]. Approximately 80% (N = 11) of the studies selected indicated that the assistance provided by CMNs was effective in relation to chronicity [28,29,31,32,34,36,37,38,39,40,41].

## 4. Discussion

The objective of this study was to analyse the scientific evidence about the care and assistance provided by CMNs to chronic patients in PHC. The main findings indicate that the assistance provided by CMNs is focused on chronic pathologies such as diabetes, arterial hypertension, COPD, coronary diseases, and behavioural health problems. Regarding the APNs’ competencies and effectiveness, the studies pointed out that the assistance provided by CMNs is effective in relation to chronicity [32].

### 4.1. Assistance Provided by CMNs to Chronic Patients and Caregivers in PHC

CMNs improved the arterial hypertension results and reduced the number of complications. Diabetes is better controlled with an integrated and multidisciplinary approach, and care management is a quality assurance component. They also assisted in reducing the glycated haemoglobin levels in diabetic patients [37,42], in addition to improving their waist circumference, Body Mass Index, and adherence to the treatment [41].

A study showed positive results in the assistance provided by CMNs to women with chronic diseases in situations of vulnerability and frailty, as well as in relation to medically complex and frail children and young individuals with chronic health problems, indicating increased satisfaction levels in the patients and their family caregivers [35].

The caregivers stated that they felt greater emotional and instrumental support through the assistance provided by CMNs, in addition to enhanced accessibility both at the in-person level and via telephone calls and home visits, which reduces psychological distress in many cases, thus providing improved safety. It is necessary to conduct studies with longer interventions led by CMNs in order to better show the assistance provided and the long-term results [15,28,31,38].

In a meta-analysis study, it was shown that CMNs ease care continuity and prevent hospital admissions. The CM model is focused on people with complex and long-term conditions (chronicity). The individuals treated indicated positive experiences and improved their quality of life, fostering self-management and personal accountability [29].

In relation to the number of urgency consultations, some studies [31,36,40] evidenced a reduction in the use of in-hospital emergency services among patients assisted by CMNs, in addition to fewer hospital admissions and increased satisfaction levels due to the care provided by CMNs, both in patients and in caregivers. The aforementioned is in line with other studies outside of this review, which suggests that patients with chronic pathologies are characterised by significantly resorting to health services, especially urgency consultations and hospital admissions, which is a reason why CMN interventions can contribute to reducing health expenses [9,15].

PHC nurses believe that the assistance provided by CMNs benefits patients and caregivers alike. However, they consider it necessary to improve the communication channels and mechanisms among them in order to achieve better interprofessional coordination between PHC nurses and CMNs. This would suppose an improvement in teamwork and, therefore, an enhancement in terms of human and material resources. Care continuity is fundamental in the nursing process: it is for this reason that CMNs should perform evaluations and follow-ups to improve care quality [23,36,43].

The Andalusian 2020–2023 Strategic Plan prioritises the assistance provided to groups of patients affected by chronic diseases with a prolonged evolution by the means of instruments that allow for proactive follow-ups at the PHC level, through care plans, objectives, and evaluations shared with the patients via useful tools such as the following: Clip-Salud, which fosters tele and video consultations and using professionals’ voicemails and emails; the App Salud Andalucía; and the development of telemedicine [24].

### 4.2. CMNs’ Competencies and Effectiveness

The assistance that CMNs provide to patients reduces the habitual complications usually presented by COPD patients, in addition to simultaneously reducing the number of hospitalisations [34]. Furthermore, significantly positive results have been evidenced in patients with chronic pathologies such as diabetes, COPD, and coronary diseases in relation to objective clinical measures, quality of life and functionality, patient satisfaction, adherence to the treatment, self-care, and the use of services. In this sense, with the Primary Care Strategy, Andalusia has initiated proactive follow-ups of patients with chronic pathologies (COPD and heart failure), strengthening the role of CMNs in relation to people with complex chronic diseases [24,32].

CMNs serve as educators, counsellors, and case managers, showing positive and effective results in telephone follow-ups and email messages. They are active participants in the process of identifying patients that require care after hospital discharge, performing a coordinating role in the process and turning out to be key actors for patient- and family-centred assistance [29,42,44].

A randomised clinical trial proposed valuing the effectiveness of a care model comprising an Internist and in-hospital Liaison Nurse, with subsequent follow-ups at the PHC level; it also reflected that this intervention was not adequate for all groups of patients. It was not efficient in patients over the age of 80; however, those aged less than 80 years old with at least three chronic diseases managed to reduce the costs in relation to consultations with urgency services and hospitalisations [39].

Through the assistance provided by CMNs in the PHC scope, improvements have been achieved in patients’ clinical conditions in relation to care quality and efficiency, as well as improvements in terms of efficacy in the use of the resources and the quality and compliance of the process. Weaknesses have also been noticed, as good-quality research studies are required in relation to the use of specific result indicators, classification of CM services, and expanded populations, offering a better-quality service that provides more customised care [45].

Two studies suggested that CMNs need special training to be able to offer effective assistance. Therefore, mandatory and continuous training is required, as well as methods to evaluate competencies and measures for ongoing control in terms of safety and quality. Regarding chronic diseases, these nurses need training in advanced practices related to chronicity. Communication skills and professional experience are also important [33]. Managers should take into account that CMNs need to have ample professional experience, knowledge about the health system and intra- and extra-hospital resources, in addition to competencies that allow them to know each patient, family, and the improvements for the health conditions in depth [44].

CMNs are nurses with advanced practice nursing (APN) competencies, which improve chronic patients’ health [31]. This is in line with other studies not discussed in this review, where CMNs have been considered as effective and efficient APNs [22,23]; in addition, it has been stated that APNs possess specialised knowledge, as well as skills in complex decision making and clinical competencies [21].

More than 80% of the studies selected in the review mentioned that, through the assistance they provide, CMNs can reduce health expenses, which supposes a benefit at the economic and political levels; it is for this reason that research studies should be encouraged to understand these costs in depth, so that this can translate into improvements in health systems at the global level. Therefore, it is important to enhance the CMN figure for assistance to be provided to chronic patients [28,29,31,32,34,36,37,38,39,40,41]. In this sense, we should take into account population aging and the increases in chronic diseases, as these imply increased health expenses in the future [1,2,6].

### 4.3. Opportunities and Barriers

Among the opportunities available, we can highlight that primary healthcare CMN interventions provide a better health literacy and self-management of care in people affected by chronic health problems [35]. This leads to better control of certain chronic diseases, including hypertension, diabetes, and chronic obstructive pulmonary disease [34,37,41,42].

Among the barriers, specific training, advanced practice competencies, and professional experience are considered as necessary for primary healthcare CMNs to provide effective and quality care for people with chronic diseases [33].

### 4.4. Added Value of this Study

The study highlights the work performed by primary healthcare CMNs through outpatient follow-up, which allows for improved healthcare and reduced healthcare costs, in addition to improving care for caregivers [31,38].

People tended by CMNs reported positive experiences and an improvement in their quality of life [29,31], as well as a decrease in the use of emergency services [31,36,40]. This study highlights the importance of interlevel coordination by the primary healthcare CMN [30].

### 4.5. Areas for Research Expansion and Suggestions for Future Developments

In relation to future research, studies on primary healthcare CMN interventions are needed to assess long-term health outcomes [28], taking into account the specific characteristics of patients [39].

Based on the results of this review, research studies on other prevalent chronic diseases should be considered [32,41].

Advanced practice profiles, such as primary healthcare CMNs, should be investigated to encourage the development of these figures [32].

Regarding the limitations of this review, one is that an assessment of the quality of the selected studies was not carried out, and another limitation is that only studies in English, Spanish, and Portuguese were included.

## 5. Conclusions

Healthcare models need to adapt to the new needs caused by the increases in complex chronic diseases, which affect people of any age and require special care from the PHC level. CMNs prepare customised management plans, evaluate social environments and lifestyles, and empower patients; all of this generates improvements in the quality of life of chronic patients and their caregivers. In addition, they have proven to be effective through ongoing assistance and interlevel and interdisciplinary coordination. Finally, we should state that new health organisation models, such as APNs (including CMNs), are required to achieve more effective and efficient results. Deeper implementation of the CMN figure should be sought, in addition to tools that allow for assessing and evaluating the work performed by these professionals.

## Figures and Tables

**Table 1:** Characteristics of the studies selected in the review.

Author, Year, and Locus	Study: Type, Methodology, and Objective	Sample (N), Gender, Age, and Period	Main Findings
Hudon et al., 2018 [28] Canada	Mixed methods. Randomised controlled trial. First and second phase: qualitative and descriptive design. To assess the effects of a case management intervention for users with chronic diseases and complex needs, in relation to psychological distress.	N = 247 patients (126 in the Intervention Group and 121 in the Control Group). Age: patients aged from 18 to 80 years old and 69 key informants. From February 2013 to January 2014.	The case management intervention reduces psychological distress, enhancing patient safety. Many patients and family caregivers stated improvements in self-control ability; however, this did not translate into greater patient activation in the controlled trial. Future studies should assess the effect of a longer intervention (1 year), as this would improve self-care.
Askerud and Condel, 2017 [29] New Zealand	Qualitative meta-synthesis. To know the patients’ experience in relation to the assistance provided by CMNs.	N = 1052 individuals sampled. Age: 55–90 years old.	Case management in charge of nurses generated high safety and confidence levels and helped the individuals to self-manage their conditions in the long term. CMNs provided professional coordination and a long-term personal relationship, easing care continuity and preventing hospital readmissions. During the last 20 years, case management has been developed as a care model for people with long-term complex chronic conditions; it is a holistic model based on strengths. The experiences of the people treated through case management were almost universally positive and helped them improve their quality of life, fostering self-management and personal accountability.
Kahn et al., 2009 [30] USA	Qualitative. To identify the problems faced by patients with behavioural health and diabetes diagnoses documented by case management nurses via telephone calls.	N = 853. Majority of women. May 2008 and January 2009.	Case management nurses address countless challenges, solving behavioural health problems and other general medical conditions. They also intervene in social and health services, coordinating patients with PHC services. This study shows how, after multiple telephone calls and email messages, the patients took the initiative of calling a case-management nurse. This nurse served as a trainer, educator, counsellor, and case manager.
Domenech-Briz et al., 2020 [31] Spain	Bibliographic review. To know the effectiveness and efficiency of CMNs in PHC.	N = 16 related articles. January and April 2020.	The assistance provided by CMNs is more effective and efficient in relation to the traditional model. The patients assisted by CMNs improved the self-management of their disease based on knowledge, and this gave rise to improvements in quality of life. Through the CMNs, the caregivers were provided emotional and instrumental support, as well as better accessibility, whether in person, via telephone calls, or in the home environment. Only one meta-analysis reflected that there was no significant evidence in relation to mortality regarding the patients assisted by CMNs. Fewer care appointments, admissions, and readmissions. Cost reductions. Greater activation of social services. Comprehensive care, easing continuity and coordination. Increased satisfaction in patients and caregivers. CMNs are APNs: they improve chronic patients’ health.
Sutherland and Hayter, 2009 [32] United Kingdom	Literature review. To assess nursing case management with one or all three main long-term chronic diseases (diabetes, COPD, and coronary diseases).	N = 108 articles (18 were included).	Significantly positive results, excellent evidence in terms of effectiveness for the impact of nursing case management in five health results: objective clinical measures; quality of life and functionality; patients’ satisfaction; adherence to the treatment; self-care; and use of the services. More research studies are required to support role development and propose a more specific intervention approach. The interventions should be maintained in time for the long-term benefits to be appreciated.
Watts and Lucatorto, 2014 [33] Boston	Literature review.	2000–2013.	Abundant scientific literature states improved assistance for patients with diabetes. No studies suggesting that a reduction in quality of life or safety problems were found. Several studies indicate cost analyses, but they fail to specify a comprehensive calculation of the ROI. Mandatory and continuous training is required to offer effective assistance as a CMN, as well as methods to evaluate competencies and measures for ongoing control in terms of safety and quality. CMNs are a cost-efficient resource for facing the medical care challenges inherent to the assistance currently provided for chronic diabetes. Adequate training for nurses about the management of chronic diseases is required. Skill and experience levels are also important.
Casas et al., 2006 [34] Spain	Quantitative. To know the efficacy of a simple, well-standardised, and integrated care intervention to prevent hospitalisations.	N = 155 patients with exacerbated COPD; 17% women.	The intervention consisted of a customised care plan shared with the PHC team, as well as access to a specialised nursing case manager through a web-based call centre. The trial shows that this intervention effectively prevents hospitalisations due to exacerbation in patients with chronic pulmonary disease.
Cook et al., 2015 [35] Florida	Quantitative. Controlled trial. To determine if a nursing case management intervention affected acquisition of a habitual care source.	N = 432. Women aged 18–60 years old. 2007–2009.	The nursing case management intervention was an efficient way to help women find a habitual care source, improving knowledge about health and the benefits and easing good-quality, cost-efficient, coordinated, and integrated assistance. Nurses employ a holistic approach, focusing their practice on health promotion; they helped the patients book appointments, learn how to handle chronic health conditions, and communicate medical care needs to health providers, social workers, and new employees.
Crane et al., 2012 [36] North Carolina	Quantitative. Randomised controlled trial with 12-month follow-up in a PHC clinic from Brazil. To examine the effectiveness of nursing case management for blood pressure control in Brazilian adults.	N = 94 (47 in the Intervention Group and 47 in the Control Group). Adults with arterial hypertension. From December 2016 to December 2017	Nursing case management can improve the effects of the arterial hypertension management strategy used in PHC. The results showed reductions in blood pressure, waist circumference, and BMI, in addition to an improvement in adherence to the treatment. The individuals from the Intervention Group had fewer hospital admissions and lower schooling levels. This model should be tested and expanded to other chronic diseases.
Gabbay R., 2013 [37] USA	Quantitative. Randomised controlled trial conducted over 2 years in 12 PHC clinics. To determine if duly training CMNs in motivational interviews would provide better results in high-risk patients with type 2 diabetes.	N = 545 patients (313 in the Control Group and 232 in the Intervention Group). From August 2006 to March 2008.	The CMNs improved blood pressure and the detection of complications. Improvements were observed in HbA1C, low-density lipoproteins, and systolic blood pressure in both groups. The scores for the depressive symptoms were better in the Intervention Group. Diabetes is better controlled with an integrated and multidisciplinary approach. Care management is considered as a core characteristic and key component of quality assurance.
Gordon et al., 2007 [38] Wisconsin	Quantitative. Up to 3 years of data before and after the patients’ enrolment in the Special Needs program were compared. To assess the impact of a Special Needs program at a tertiary-level care centre that partners with families and PHC physicians to ensure uninterrupted in-hospital and outpatient care and help provide medical homes.	N = 227. Medically complex and frail children and young individuals, with a broad range of chronic disorders. From 1 July 2002, to June 2005.	This new association model between tertiary-level assistance and PHC was effective in improving the care provided to the patients, their health, and the cost reductions achieved. The key interventions were as follows: collaboration with the family; familiarity with the child’s pathology; close participation during the hospitalisation periods; and proactive outpatient care. The patients and families indicated an increase in their satisfaction level.
Lanzeta et al., 2016 [39] Spain	Quantitative. Clustered randomised clinical trial. To perform a cost–benefit analysis of an integrated care model comprising a designated Internist and a hospital Liaison Nurse for patients with multiple morbidities, when compared to a conventional reactive health system.	N = 140 patients. 70 patients in each group. It lasted 1 year: from April 2011 to February 2012.	The intervention was not efficient. The subgroup comprising patients aged less than 80 years old with at least 3 clinical categories saved 89% of the costs in the simulations. The intervention was not suitable for all patients; the subgroup analysis allowed for identifying a more specific target population that should be analysed in future studies.
Mallitt et al., 2016 [40] Australia	Quantitative (before and after comparison). To determine the results of the HealthOne Mount Druitt (HOMD) coordinated care program, with coordination provided by liaison nurses.	N = 125 participants. Majority of women (58.4%). 68.4 years old (from 17 to 93).	The HOMD was certainly positive: more comprehensive planning for the clients (82%); closer work links with other organisations (76%); better relationships with health workers (73%); and enhanced coordination of services for patients with multiple needs (75%). Showed qualitative evidence that a coordinated care intervention (HealthOne) improves patients’ health results. Greater integration of services, coordination, flexibility, and care continuity. Lesser use of hospital emergency services and greater access to a combination of allied health services in the community.
Mattei et al., 2019 [41] Brazil	Quantitative. Randomised controlled trial with 12-month follow-up in a PHC clinic from Brazil. To examine the effectiveness of nursing case management for blood pressure control in Brazilian adults.	N = 94 (47 in the Intervention Group and 47 in the Control Group). Adults with arterial hypertension. From December 2016 to December 2017.	Nursing case management can improve the effects of the arterial hypertension management strategy used in PHC. The results showed reductions in blood pressure, waist circumference, and BMI, in addition to an improvement in adherence to the treatment. The individuals from the Intervention Group had fewer hospital admissions and lower schooling levels. This model should be tested and expanded to other chronic diseases.
Watts and Sood, 2016 [42] USA	Quantitative. To determine the efficacy of a case management program to improve the qualifications of diabetes educators certified in case management nursing.	N = 15,636 patients during a 10-year period. From 1 July 2004, to 7 July 2014.	The intervention led by case management nurses showed a significant reduction in glycated haemoglobin.

CMN: case management nurse. PHC: primary healthcare. COPD: chronic obstructive pulmonary disease. BMI: Body Mass Index. HOMD: HealthOne Mount Druitt. APN: advanced practice nurse. Source: The authors.

## Data Availability

Not additional data available.
